# Relationship Between Resilience, Social Support, Existential Well-Being and Negative Emotions in Cervical Cancer Patients: a Mediation Analysis

**DOI:** 10.7150/jca.91260

**Published:** 2024-04-29

**Authors:** Yaomei Ma, Shiyue Chen, Hanyu Dong, Ruimeng Guo, Ruoyan Liu, Juan Xie, Zhuoyu Sun

**Affiliations:** 1 Department of Gynecological Oncology, Tianjin Medical University Cancer Institute & Hospital, Tianjin, China.; 2 Department of Epidemiology and Biostatistics, School of Public Health, Tianjin Medical University, Tianjin, China.; 3 Tianjin Key Laboratory of Environment, Nutrition and Public Health, Tianjin, China.; 4 Center for International Collaborative Research on Environment, Nutrition and Public Health, Tianjin, China.; 5 Department of Gynecological Oncology, The Second Hospital of Tianjin Medical University, Tianjin, China.

**Keywords:** resilience, social support, existential well-being, negative emotions, cervical cancer, mediation analysis

## Abstract

**Background:** The patients of cervical cancer have more negative emotions and lower quality of life. The aim of this study was to explore the relationships between existential well-being (EWB), social support, resilience, negative emotions in patients with cervical cancer, and to examine whether resilience mediates the associations between EWB or social support and negative emotions.

**Material and methods:** This study enrolled patients with cervical cancer who were treated at the Tianjin Medical University Cancer Institute and Hospital in China during 2012-2019. The Hospital Anxiety and Depression Scale (HADS), the Resilience Scale of 14 items (RS-14) and the McGill Quality of Life Questionnaire (MQOL) were utilized to assess patient's anxiety, depression, resilience, social support and EWB via telephone. Spearman's correlation analyses were used to assess bivariate correlations, and mediation analyses were applied to examine whether resilience mediated the relationship between social support or EWB and negative emotions.

**Results:** A total of 150 (92.0%) out of 163 eligible patients completed the questionnaires. EWB and social support were negatively correlated with anxiety (*r*=-0.560 and *r*=-0.561) and depression (*r*=-0.508 and *r*=-0.526), and positively correlated with resilience (*r*=0.691 and *r*=0.652). Resilience was negatively associated with anxiety (*r*=-0.545) and depression (r=-0.505). Negative direct effects of social support on anxiety and EWB on anxiety and depression were statistically significant (*P*<0.05). Resilience played a partial mediating role in the relationship between EWB and depression (*β*=-0.085, 95%*CI*: -0.150 to -0.020), accounting for 37.12% of the total effect. It also served as a partial mediator in the association between EWB and anxiety (*β*=-0.061, 95%*CI*: -0.107 to -0.015), explaining 34.46% of the overall effect. Additionally, resilience partially mediated the connection between social support and depression (*β*=-0.173, 95%*CI*: -0.312 to -0.053), explicating 57.48% of the total effect.

**Conclusions**: A combination of existential, supportive and resilient interventions may help reduce psychological distress and improve quality of life among cervical cancer patients, thereby promoting both physical and psychological health.

## Introduction

Cervical cancer is a prevalent and serious disease, ranking as the fourth most frequent malignancy in women and the fourth leading cause of cancer-related deaths worldwide, with an estimated 604,127 new cases and 341,831 deaths in 2020^1^. The survival duration of women with cervical cancer has increased due to advances in treatment technology and the widespread implementation of screening programs. Consequently, there is an increasing focus on the quality of life and prognosis of cervical cancer patients. Several studies have found that negative emotions were related to poorer cancer survival and higher cancer-specific mortality in cervical cancer patients, independents of tumor characteristics and treatment modality 2, 3. Due to the specificity of the cancer site and HPV infections, cervical cancer can adversely affect patients' fertility, sexual function, self-identity, self-image and self-esteem 4. Such adverse impacts can exacerbate negative emotions (including anxiety and depression) in cervical cancer, underscoring the importance of monitoring and promoting the psychological health of these patients.

Resilience, which is considered one of the positive psychological factors, is a set of capacities or processes that enable individuals to successfully cope with adversities and maintain their psychological and physical well-being 5. Greater resilience is associated with enhanced problem-solving abilities, as well as increased creativity and self-efficacy to respond positively to adversities. Studies have shown that breast cancer patients who participated in resilience training programs experienced effective improvements in anxiety and depression 6, 7. This may be attributed to the fact that resilience training can provide individuals with more protective factors, such as internal factors including comprise cognitive, emotional, spiritual and social factors 8.

Existential well-being (EWB) is a dimension of spiritual well-being that pertains to an individual's subjective sense of meaning and purpose in life. Studies have shown that individuals with higher levels of EWB exhibited better physical, role, emotional, conscious, and social functioning, as well as fewer symptoms 9. On the contrary, those experiencing existential distress experienced a diminished quality of life, negative emotions, demoralization, and even suicidal ideation 10. The presence of meaning and purpose in life has been found to provide hope and positively influence patient survival 11. Additionally, a negative association has been observed between spiritual well-being and negative emotions, while a positive correlation has been found between spiritual well-being and positive adaptation to cancer 12. Given these findings, it appears that the experience of meaning and existential concerns play a particularly significant role in the context of cancer patients 13.

Social support refers to the provision of assistance, care, emotional support, and positive regard by others to an individual in need 14. Research has suggested that an increased risk of cancer may be associated with inadequate social support 15. Conversely, higher levels of perceived social support have been found to have a direct or indirect effect on reducing negative emotions, through the promotion of self-esteem, hope and resilience in patients with lung and prostate cancer 16, 17. Moreover, patients with colon, breast and gynecologic cancer have reported a positive correlation between perceived social support and spiritual well-being and positive mental adjustment 18, 19. Additionally, a robust social support system has been shown to mitigate psychological stress following trauma and promote resilience.

Cervical cancer is a traumatic event that has a significant impact not only on the physical health but also on the psychological well-being of patients. The treatment of cervical cancer, along with its associated side effects, may cause issues with gender identity and changes in body image, which can hinder the patients' ability to resume satisfactory sexual functioning 4. Moreover, cervical cancer is often stigmatized in society due to its association with HPV 20, which may result in patients feeling ashamed and self-blaming, particularly when concerning the opinions of those around them, such as their partner. These factors can lead to negative emotions, decreased self-worth, and heighted worry and fear concerning interpersonal relationships. Although studies have indicated that social support and EWB can alleviate negative emotions in patients with other types of cancer, few studies have explored the relationship between EWB or social support and negative emotions in cervical cancer patients, and whether resilience affected the relationship was rarely discussed. Hence, the aim of this study was to explore the relationship among EWB, social support, resilience and negative emotions in patients with cervical cancer, and to examine whether resilience mediates the associations between EWB, social support and negative emotions. Our research findings aim to provide theoretical evidence for enhancing the mental health and health related quality of life of cervical cancer patients.

## Materials and Methods

### Study design and eligibility

Medical records were extracted for a retrospective cohort of all cervical cancer patients admitted to the Tianjin Medical University Cancer Institute and Hospital in China during 2012-2018. The inclusion criteria consisted of patients diagnosed with cervical malignant tumors confirmed by pathological diagnosis, patients who have completed full treatment at least one year prior to enrollment, patients aged between 18 and 85 years old, and those with complete medical records available. Exclusion criteria included patients with local recurrence or distant metastasis, patients with concomitant other tumors, patients with a history of psychological disorders such as anxiety or depression before cancer diagnosis, patients with communication and understanding barriers, and those with mental or intellectual disabilities.

Eligible patients were contacted via telephone, whose numbers were obtained from the medical records. After explaining the study, research assistants obtained patients' oral informed consent before administering a validated structured questionnaire. Clinical data were retrieved from medical records. The present study was approved by the Institutional Ethics Review Board of Tianjin Medical University Cancer Institute and Hospital.

### Outcomes and variables

The assessment of negative emotions in this study was conducted using the Hospital Anxiety and Depression Scale (HADS) 21, which is comprised of two subscales: anxiety (HADS-A) and depression (HADS-D). Each subscale consists of seven items measured on a 4-point Likert scale ranging from 0 to 3. The total score for each subscale ranges from 0 to 21, with higher scores indicating more severe symptoms of anxiety and depression. In Chinese cancer patients, the HADS has demonstrated high levels of reliability and validity, with a Cranach's alpha of 0.855 for HADS-A and 0.879 for HADS-D 22.

The level of resilience was evaluated using the Resilience Scale of 14 items (RS-14) 23. Responses for the 14 items were graded on a 7-point Likert scale ranging from “1” (strongly disagree) to “7” (strongly agree). The total score ranged from 14 to 98, with higher scores indicating higher levels of resilience. The Chinese version of RS-14 has also been used in Chinese cancer patients, with high levels of reliability and validity confirmed (Cranach's alpha of 0.93 for overall RS-14 and the factor analysis explained 61% of the variance, indicating acceptable validity) 24.

Social support and EWB were assessed using the two subscales from the McGill Quality of Life Questionnaire (MQOL) 25. MQOL includes four subscales: physical, psychological, EWB, and social support, of which the subscales of EWB (consisting of 5 items) and social support (consisting of 3 items) were utilized. The items were rated on a scale of 1 to 10, and the total score was calculated as the mean of all item scores, with a higher score indicating better well-being. The Chinese version of the MQOL demonstrated high reliability and validity in cancer patients, with a Cranach's alpha of 0.917 for support and 0.857 for EWB. Factor analysis explained 74.83% of the variance, indicating acceptable validity) 26.

Demographic characteristics (including age, height, weight, living place, education level, marital status, occupational condition, family income, smoking status, drinking status, physical activity, medical insurance and family relationships) and clinical characteristics (including stage, histology, treatment and diagnosis of time) were also collected in this study. Body mass index (BMI) was calculated by dividing weight (kilogram) by the square of height (meter). BMI was categorized using the cut-points established by the Bureau of Disease Control of Ministry of Health in 2003 for Chinese adults: underweight (<18.5 kg/m^2^); normal weight (18.5-23.9 kg/m^2^); overweight (24.0-27.9 kg/m^2^) and obese (≥28.0 kg/m^2^) 27.

### Statistical analysis

Descriptive statistics were utilized to summarize all variable. The Mann-Whitney *U* test or Kruskal-Wallis *H* test was employed to examine whether there were differences in the mediator (resilience) and dependent variables (anxiety and depression) based on demographic and clinical characteristics. Spearman's correlation analyses were used to assess bivariate correlations among resilience, anxiety, depression, social support and EWB. Mediation analyses were applied to examine whether resilience mediated the relationship between social support or EWB and negative emotions 28, 29. As part of the mediation analysis, multiple linear regression models were applied to investigate total and direct effects. Both Model 1 [Y ~ X + ... (adjusted variables) ] and Model 2 [Y ~ M + X + ... (adjusted variables) ] were utilized for the analysis of indirect effects. Here, Y represents anxiety or depression, X represents social support or EWB, M represents resilience, and adjusted variables, including age, living place, education level, family income, physical activity and family relationships, were incorporated for adjustment purposes. The 95% confidence interval *(CI)* of indirect effects was estimated using a bootstrapping method with 5000 resamples. Mediation was confirmed if the bias-corrected 95%*CI* did not include zero. Demographic and clinical variables that affect resilience and anxiety or depression were entered as control variables in the mediation model. SPSS, version 23.0 (IBM Inc., Chicago, IL, USA) was used for all analyses, and mediation analyses were conducted using the SPSS PROCESS 3.4 macro (Model 4) 28. Statistical significance was considered at *P*<0.05.

## Results

### Description and comparison of demographic and clinical characteristics

Out of the total of 163 eligible cervical cancer patients, 150 completed the questionnaires, resulting in a response rate of 92.0%. The mean age of the sample was 52.2±9.8 years, ranging from 29 to 74 years. The majority of the patients were overweight or obese (56.7%), married or in a common-law relationship (87.3%), retried or unemployed (70.7%), and resided in urban areas (62.7%). About 60% of them reported a monthly family income of ≤5000 RMB, and 59.3% reported never or occasionally engaging in physical activity. More than 90% of the participants had medical insurance, and they reported being satisfied with their family relationships. Participants who resided in urban areas, those who often engaged in physical activities and those reported being satisfied with family relationships had significantly higher levels of resilience and lower levels of anxiety and depression (*P*<0.05). Additionally, participants with higher levels of education and family income reported significantly lower levels of depression (*P*<0.05) (see Table 1).

Clinical characteristics of the participants were presented in Table 2. Of the patients included in the study, 58.0% were diagnosed with stage Ⅰ cervical cancer, while 54.0% were diagnosed with squamous cell carcinoma. The majority of the patients underwent surgical intervention, either alone or in combination with adjuvant chemo-radiotherapy, accounting for 74.0% of the total patients. The median duration of follow-up for all patients was 34 months (IQR: 23-54 months). No significant differences in levels of resilience, anxiety and depression were observed among patients with varying clinical characteristics.

### Relationships among negative emotions, social support, EWB and resilience

Results of the Spearman's correlation analysis (Table 3) revealed negative correlations between anxiety and social support (*r*=-0.561, *P*<0.001), EWB (*r*=-0.560, *P*<0.001) and resilience (*r*=-0.545, *P*<0.001). Similar associations were observed between depression and social support (*r*=-0.562, *P*<0.001), EWB (*r*=-0.508, *P*<0.001) or resilience (*r*=-0.505, *P*<0.001). Furthermore, the study found significant positive associations between resilience and social support (*r*=0.691, *P*<0.001), as well as EWB (*r*=0.652, *P*<0.001).

### Mediating effect of resilience in the relationship between social support, EWB and negative emotions

**Figure 1:**
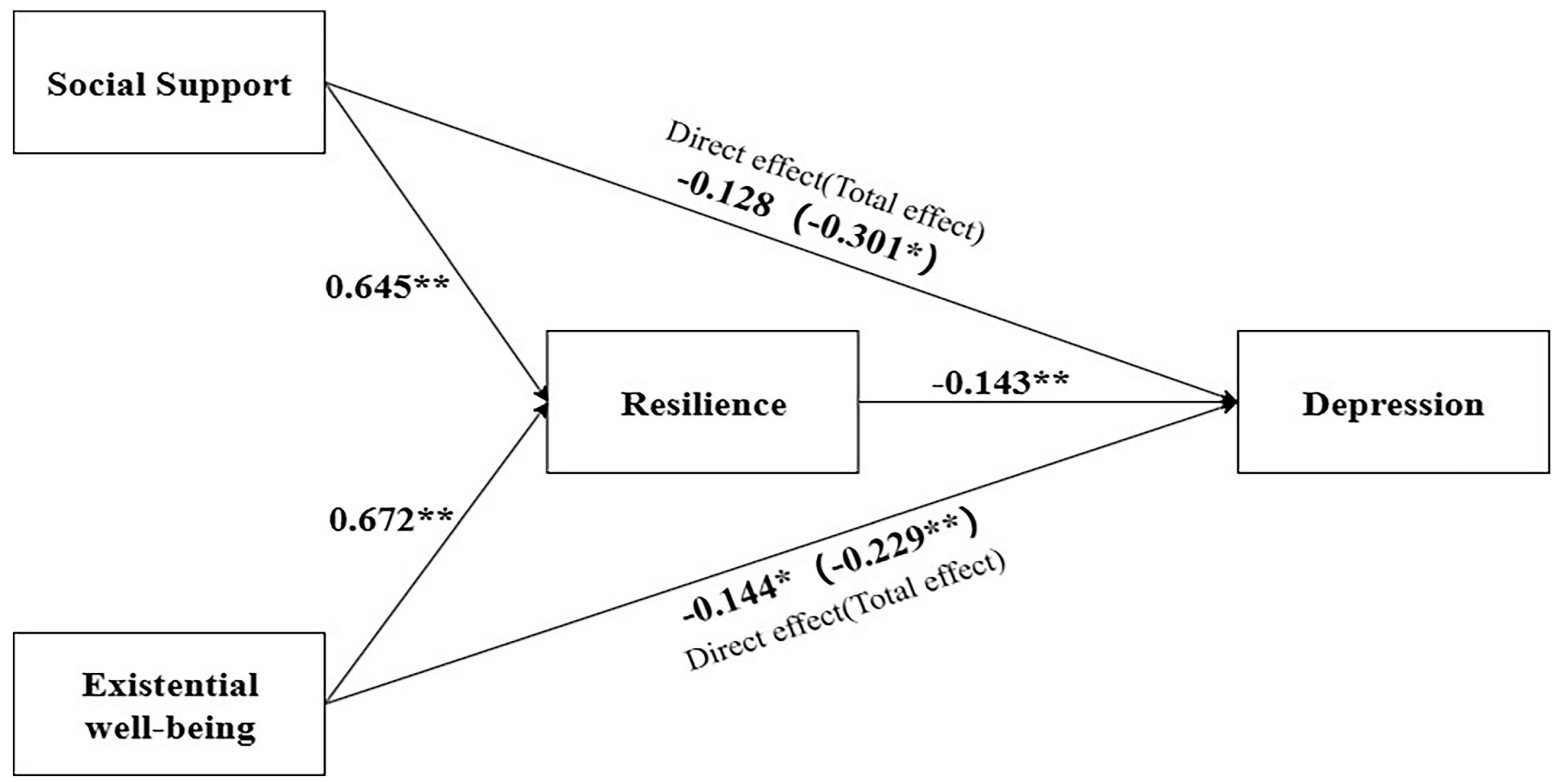
** Mediating effects^ a^ of resilience on the relationship between social support or existential well-being and depression (N=150).** ^a^ Both Model 1 [Y ~ X + ... (adjusted variables) ] and Model 2 [Y ~ M + X + ... (adjusted variables) ] were utilized for the analysis of indirect effects, with Y indicating depression, X indicating social support or existential well-being, M indicating resilience and adjusted variables including age, living place, education level, family income, physical activity and family relationships. ^*^P<0.05, ^**^P<0.001.

**Figure 2:**
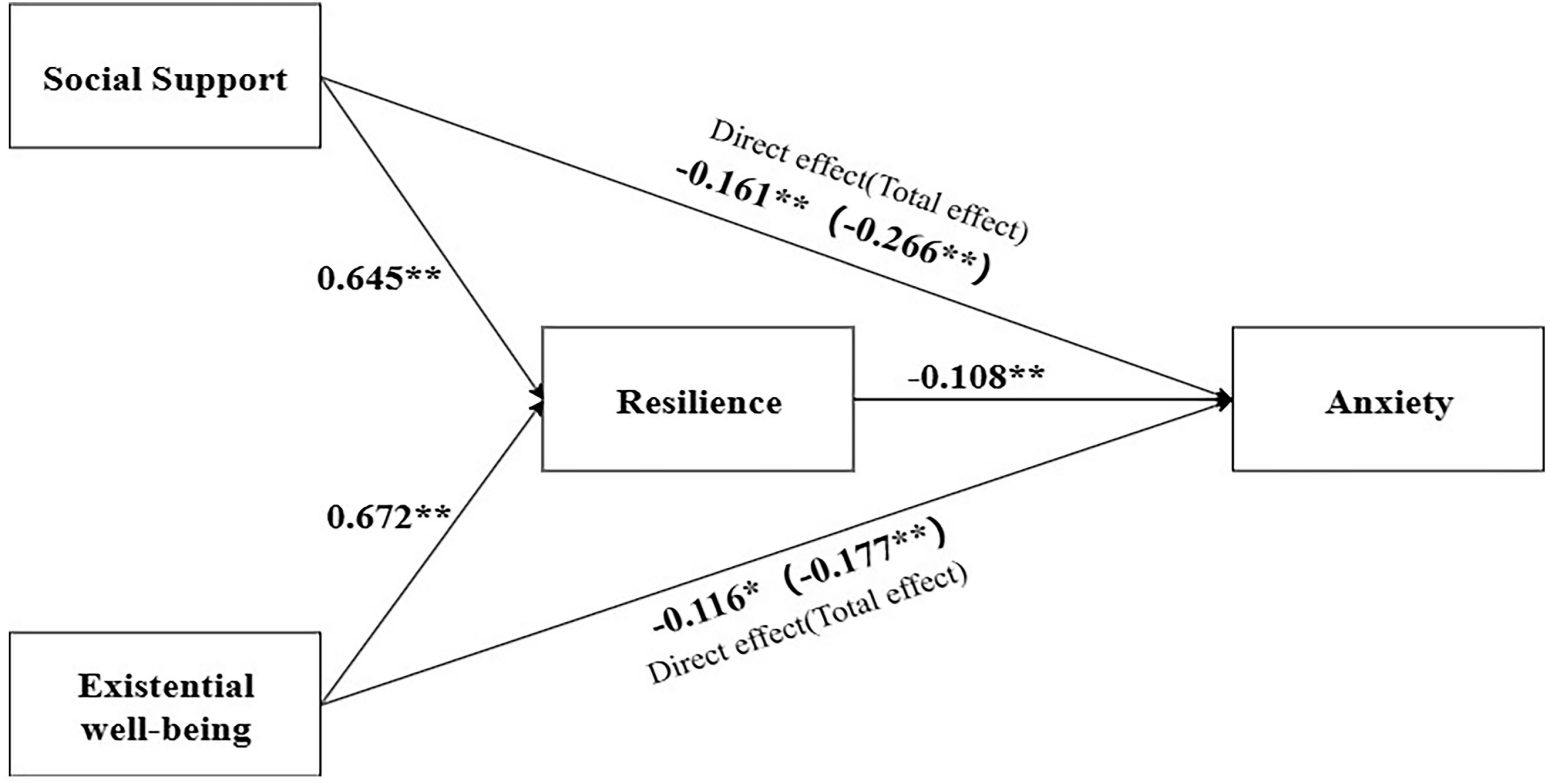
** Mediating effects ^a^ of resilience on the relationship between social support or existential well-being and anxiety (N=150).** ^a^ Both Model 1 [Y ~ X + ... (adjusted variables) ] and Model 2 [Y ~ M + X + ... (adjusted variables) ] were utilized for the analysis of indirect effects, with Y indicating anxiety, X indicating social support or existential well-being, M indicating resilience and adjusted variables including age, living place, physical activity and family relationships. ^*^P<0.05, ^**^P<0.001.

Models 1 and 2, expressed as [Y ~ X + ... (adjusted variables)] and [Y ~ M + X + ... (adjusted variables)], respectively, were employed to assess indirect effects. Here, Y denotes anxiety or depression, X represents social support or EWB, M signifies resilience within the scope of the analysis and adjusted variables were included for adjustment purposes. The total effect of social support (or EWB) on negative emotions were statistically significant (*P*<0.05) (Table 4). Specially, there were negative direct effects of social support on anxiety (*β*=-0.161, 95%*CI*=-0.247 to -0.074) and depression (*β*=-0.128, 95%*CI*=-0.241 to -0.015) and of EWB on anxiety (*β*=-0.116, 95%*CI*=-0.172 to -0.061) and depression (*β*=-0.144, 95%*CI*=-0.215 to -0.074). Notably, resilience partially mediated the relationship between EWB and depression (*β*=-0.085, 95%*CI*: -0.150 to -0.020), explaining 37.12% of the overall effect. It also served as a partial mediator in the connection between social support and depression (*β*=-0.173, 95%*CI*: -0.312 to -0.053), accounting for 57.48% of the total effect, as shown in Table 4 and Figure 1. Meanwhile, resilience played a partial mediating role in the association between EWB and anxiety (*β*=-0.061, 95%*CI*: -0.107 to -0.015), explaining 34.46% of the total effect, as shown in Table 4 and Figure 2. However, resilience was not found as a mediator between social support and anxiety, as shown in Table 4 and Figure 2.

## Discussion

The study findings indicated that EWB and social support demonstrated a negative correlation with anxiety and depression, while displaying a positive correlation with resilience. Furthermore, resilience exhibited negative associations with anxiety and depression. Based on the observed correlations, the study conducted mediation analyses, revealing that negative direct effects of social support (or EWB) on both anxiety and depression were statistically significant. Additionally, resilience was found to be a partial mediator in the relationship between EWB and anxiety (or depression), as well as in the association between social support and depression.

In the current study, social support was negatively correlated with negative emotions and demonstrated a direct impact on anxiety and depression. The phenomenon may be elucidated through the origin of support in cervical cancer patients. Specifically, the present study discovered that participants living in urban areas and satisfied with their family relationships reported lower levels of depression and anxiety. Support from family members, friends, or the community can satisfy cervical cancer patients' belongingness, love and respect, thereby reducing feelings of loneliness, dissatisfaction, and negative emotions 31, 32. Additionally, a cervical cancer patient's social psychology is significantly influenced by their social network. Misconception about HPV transmission can lead to misunderstanding about cervical cancer among the public and patients alike, causing patients to feel isolated and ashamed. The lack of understanding and support from people around them may exacerbate patients' negative emotions. Therefore, patients who perceived stronger support exhibit fewer symptoms of anxiety and depression.

Similar findings were found for EWB in the present study. EWB had a negative correlation with anxiety and depression, and exhibited a direct impact on negative emotions. These findings were in line with those of Kim *et al.* 33, who confirmed that cervical cancer patients with lower EWB were at a greater risk of developing anxiety (*OR*=3.1) and depression (*OR*=9.2). This finding may be explained by the notion that possessing higher levels of EWB can promote learning and the development of more effective emotion regulation skills 34. Meanwhile, higher EWB has been associated with fostering hope in patients and positively influencing patient survival 35. A strong sense of life purpose can enable patients to better cope with life and reduce uncertainty, thereby alleviating negative emotions.

The present study demonstrated that resilience was negatively associated with anxiety and depression. This result was consistent with those of a previous systematic review, which showed that higher resilience scores in adult cancer patients were linked to lower scores for anxiety and depression and higher scores for quality of life 8. This outcome may be related to the fact that resilient patients possess a greater number of intrinsic and extrinsic protective factors and are better equipped to utilize them to their fullest potential. Harms *et al.* similarly confirmed that higher personal and psycho-social (from family and peers) protective factors for resilience were associated with fewer symptoms of depression and anxiety in cancer patients 36. Moreover, the resilience framework posits that when individuals encounter adversity, they draw upon their inherent strengths, interact with protective and risk factors in the external environment, and adapt and cope resiliently. Our study found a positive correlation between EWB (or social support) and resilience, indicating that higher levels of EWB and social support may serve as protective factors for resilience. In addition, our research findings revealed resilience to mediate the relationship between social support (or EWB) and depression in cervical cancer patients. This outcome aligns with the aforementioned resilience framework.

In our study, resilience was found to partially mediate the association between social support and depression. Consistent with our study, Hu *et al.* reported that resilience was a mediator in the relationships between subjective support, utilization of support and depression in lung cancer patients 37. Cervical cancer patients might benefit from information support to enhance their knowledgeable of the disease and physical changes, while emotional support, particularly from a partner, may provide comfort during the illness. Thus, social support can be considered a protective factor in the external environment that assists patients in adapting and adjusting as needed. Resilient individuals tend to actively seek help from others, utilize social resources positively, and develop effective coping skills 38. Therefore, patients with higher social support may improve resilience and reduce depression.

Our finding indicated that resilience played a partial mediating role in the relationship between EWB and depression, exhibiting a mediating effect of 37.12%. Additionally, it was observed to partially mediate the association between EWB and anxiety, demonstrating a mediating effect of 34.46%. EWB can be considered as a personal resource, and prior research has found that individuals with higher levels of resilience, as well as a strong sense of purpose and mastery of life, were less likely to experience psychiatric disorders following exposure to trauma compared to those without such characteristics 37. Despite the disruption to life goals and responsibilities that may accompany a diagnosis of cervical cancer, patients with strong EWB can adapt and redirect their objectives according to their current circumstances, consistent with the construct of resilience. This result was also confirmed in a randomized controlled trial 39, which indicated that interventions aimed at promoting existential and mindfulness-based practices were effective in reducing depressive symptoms after 8 weeks, with effects similar or superior to those of mindfulness-based training alone, which was a type of resilience training 39. It should be noted that while resilience may account for some of the association between EWB and depression/anxiety, other factors, such as mastery of life, emotional regulation skills and hope, may also contribute to the relationship mentioned above.

Cervical cancer is regarded as a significant adversity for women, with potential long-term effects on patients' lives and psychology, even after treatment. Our results highlighted the importance of effective coping strategies for cervical cancer patients, particularly for those with lower levels of social support and EWB, who were at greater risk of experiencing anxiety and depression. These findings underlined the value of implementing multiple interventions, such as existential interventions, support programs, and resilience training, to enhance mental health outcomes in cancer patients.

In light of our results, it may be beneficial to combine existential interventions and supportive-expressive programs with resilience training as a potential approach to effectively reduce depression in cervical cancer patients. This approach represents a promising new direction for the development of comprehensive interventions aimed at improving mental health outcomes among cancer patients.

This is one of few studies that have examined the relationship between resilience and various factors, including EWB, social support and negative emotions, in patients with cervical cancer. Our findings provided a theoretical foundation for customized psychological interventions to enhance the mental health and quality of life of individuals with cervical cancer who are undergoing treatment and rehabilitation. However, this study had several limitations that must be considered. Firstly, we were unable to establish causal relationships among EWB, social support, resilience, anxiety and depression. Additionally, confounding bias and self-reporting bias may have affected the results. Furthermore, the participants were drawn from a single hospital, and as such, the sample might not be representative of the target population. Therefore, the results of this study need to be further verified in large prospective cohort studies.

## Conclusion

Our study showed that resilience played a mediating role in the relationship between EWB (or social support) and depression, indicating the importance of resilience in the implementation of existential and supportive interventions. Providing patients with such interventions may not only enhance their EWB and social support but also boost their resilience and reduce negative emotions. Therefore, psychological health providers should consider employing a combination of existential, supportive and resilient interventions to alleviate psychological distress in patients undergoing treatment and rehabilitation, so as to promote their overall physical and psychological well-being.

## Figures and Tables

**Table 1:** Median scores and IQR of resilience, anxiety and depression in participants with different demographic characteristics (n=150)

Variable	N (%)	Resilience	Anxiety	Depression
Age (years)^ a^				
≤50	59 (39.3)	88.0 (77.0-98.0)	2.0 (2.0-4.0)	2.0 (0.0-4.0)
>50	91 (60.7)	88.0 (79.0-97.0)	1.0 (0.0-3.0)	2.0 (0.0-4.0)
BMI (kg/m^2^)^b^				
Underweight	3 (2.0)	75.0 (61.0-93.0)	5.0 (3.0-9.0)	5.0 (0.0-11.0)
Normal weight	62 (41.3)	93.0 (79.0-98.0)	1.0 (0.0-3.0)	2.0 (0.0-3.0)
Overweight	63 (42.0)	87.0 (79.0-94.0)	1.0 (0.0-3.0)	3.0 (0.0-4.0)
Obese	22 (14.7)	91.0 (78.0-97.0)	1.5 (0.0-5.0)	1.0 (0.0-4.0)
Place of residence^ a^				
Urban	94 (62.7)	91.0 (82.0-98.0)^*^	0.0 (0.0-3.0)^*^	1.0 (0.0-3.0)^*^
Rural	56 (37.3)	84.0 (70.5-95.5)^*^	2.0 (0.0-5.0)^*^	3.0 (0.5-8.0)^*^
Education level^ a^				
Primary school or Middle school	78 (52.0)	87.0 (75.0-98.0)	1.5 (0.0-4.0)	3.0 (0.0-7.0)^*^
High school or above	72 (48.0)	90.5 (81.5-96.5)	1.0 (0.0-3.0)	1.0 (0.0-3.0)^*^
Marital Status^ a^				
Married/Common-law	131 (87.3)	88.0 (79.0-97.0)	1.0 (0.0-3.0)	2.0 (0.0-4.0)
Divorced/single/separated/ widowed	19 (12.7)	88.0 (75.0-95.0)	2.0 (0.0-3.0)	2.0 (1.0-5.0)
Employment status^ a^				
Full-time/part-time	44 (29.3)	93.0 (80.5-98.0)	1.0 (0.0-3.0)	1.0 (0.0-3.0)
Retried/unemployed	106 (70.7)	87.0 (79.0-96.0)	1.0 (0.0-3.0)	3.0 (0.0-5.0)
Family income (RMB/month)^ a^				
≤5000	90 (60.0)	85.0 (79.0-97.0)	1.0 (0.0-4.0)	3.0 (1.0-5.0)^*^
>5000	60 (40.0)	91.0 (82.0-98.0)	1.0 (0.0-3.0)	1.0 (0.0-3.0)^*^
Smoking Status^ a^				
Never	133 (88.7)	88.0 (79.0-97.0)	1.0 (0.0-3.0)	2.0 (0.0-4.0)
Ever/current smoker	17 (11.3)	89.0 (79.0-96.0)	2.0 (0.0-5.0)	3.0 (1.0-6.0)
Drinking Status^ a^				
No	130 (86.7)	90.0 (80.0-97.0)	1.0 (0.0-3.0)	2.0 (0.0-4.0)
Yes	20 (13.3)	83.0 (72.5-93.5)	2.0 (0.5-3.0)	1.0 (0.5-4.0)
Physical Activity^ a^				
Never/Occasionally	89 (59.3)	85.0 (75.0-95.0)^*^	2.0 (0.0-5.0)^*^	3.0 (0.0-5.0)^*^
Often	61 (40.7)	92.0 (84.0-98.0)^*^	0.0 (0.0-2.0)^*^	1.0 (0.0-3.0)^*^
Medical Insurance^ a^				
Yes	140 (93.3)	88.5 (79.5-97.0)	1.0 (0.0-3.0)	2.0 (0.0-4.0)
No	10 (6.7)	80.0 (65.0-93.0)	2.5 (0.0-5.0)	3.5 (0.0-9.0)
Family Relationship^ a^				
Satisfied	140 (93.3)	90.0 (80.5-98.0)^*^	1.0 (0.0-3.0)^†^	2.0 (0.0-3.0)^†^
Unsatisfied	10 (6.7)	73.0 (65.0-82.0)^*^	5.5 (3.0-7.0)^†^	7.5 (5.0-12.0)^†^

IQR, interquartile range; BMI, Body mass index ^*^P<0.05, ^†^*P*<0.001. a* p*-value for Mann-Whitney *U*-test. b *p*-value for Kruskal-Wallis test.

**Table 2:** Median scores and IQR of resilience, anxiety and depression in participants with different clinical characteristics (n=150)

Variable	N (%)	Resilience	Anxiety	Depression
Stage (FIGO)				
Ι	87 (58.0)	89.0 (79.0-97.0)	1.0 (0.0-3.0)	1.0 (0.0-4.0)
Ⅱ	46 (30.7)	88.0 (79.0-98.0)	1.0 (0.0-4.0)	3.0 (0.0-3.0)
Ⅲ	8 (5.3)	84.5 (76.5-92.5)	1.5 (0.5-3.0)	2.0 (0.5-5.0)
Ⅳ	9 (6.0)	91.0 (71.0-94.0)	0.0 (0.0-2.0)	3.0 (0.0-7.0)
Histology				
Squamous cell carcinoma	81 (54.0)	88.0 (81.0-97.0)	1.0 (0.0-3.0)	2.0 (0.0-3.0)
Adeno carcinoma	18 (12.0)	94.0 (85.0-98.0)	1.0 (0.0-6.0)	1.0 (0.0-4.0)
Adenosquamous carcinoma	51 (34.0)	92.0 (84.0-98.0)	1.0 (0.0-3.0)	3.0 (0.0-3.5)
Treatment modality				
Surgery alone	53 (35.3)	88.0 (79.0-97.0)	1.0 (0.0-3.0)	2.0 (0.0-5.0)
Chemoradiotherapy	39 (26.0)	91.0 (81.0-98.0)	1.0 (0.0-3.0)	2.0 (0.0-3.0)
Surgery plus adjuvant chemoradiotherapy	58 (38.7)	87.0 (79.0-97.0)	2.0 (0.0-4.0)	3.0 (0.0-4.0)
Follow-up time after diagnosis (months)				
≤12	4 (2.7)	95.0 (87.0-96.5)	1.0 (0.0-4.0)	0.5 (0.0-4.5)
12-36	80 (53.3)	90.0 (82.0-97.5)	1.0 (0.0-3.5)	2.0 (0.0-4.0)
36-60	31 (20.7)	87.0 (81.0-98.0)	1.0 (0.0-4.0)	2.0 (0.0-5.0)
>60	35 (23.3)	82.0 (71.0-96.0)	1.0 (0.0-2.0)	2.0 (1.0-4.0)

FIGO, International Federation of Gynecology and Obstetrics.

**Table 3:** Bivariate Spearman's correlations among anxiety, depression, social support, existential well-being and resilience

	Median (IQR)	1	2	3	4
1. Anxiety	1.0 (0.0-3.0)				
2. Depression	2.0 (0.0-4.0)	0.528*			
3. Social Support	9.3 (8.0-10.0)	-0.561*	-0.526*		
4. Existential well-being	9.0 (7.4-9.8)	-0.560*	-0.508*	0.789*	
5. Resilience	88.0 (79.0-97.0)	-0.545*	-0.505*	0.691*	0.652*

IQR, interquartile range. ^*^P<0.001.

**Table 4:** Mediating effect of resilience in the relationship between social support or EWB and anxiety or depression

	Effect	SE	*P*-value	LLCI^c^	ULCI^c^
**Social Support→Resilience→Anxiety^a^**					
Total effect	-0.266	0.036	<0.001	-0.336	-0.195
Direct effect	-0.161	0.044	<0.001	-0.247	-0.074
Indirect effect	-0.105	0.042		-0.195	0.027
**Existential well-being→ Resilience→Anxiety^a^**					
Total effect	-0.177	0.022	<0.001	-0.221	-0.134
Direct effect	-0.116	0.028	0.001	-0.172	-0.061
Indirect effect	-0.061	0.024		-0.107	-0.015
**Social Support→Resilience→Depression^b^**					
Total effect	-0.301	0.048	<0.001	-0.395	-0.207
Direct effect	-0.128	0.057	0.027	-0.241	-0.015
Indirect effect	-0.173	0.067		-0.312	-0.053
**Existential well-being→ Resilience→Depression^b^**					
Total effect	-0.229	0.028	<0.001	-0.285	-0.174
Direct effect	-0.144	0.036	<0.001	-0.215	-0.074
Indirect effect	-0.085	0.033		-0.150	-0.020

SE, standard error; LLCI, lower-level confidence interval; ULCI, upper-level confidence interval. **^a^** Both Model 1 [Y ~ X + ... (adjusted variables) ] and Model 2 [Y ~ M + X + ... (adjusted variables) ] were utilized for the analysis of indirect effects, with Y indicating anxiety, X indicating social support or existential well-being, M indicating resilience and adjusted variables including age, living place, physical activity, and family relationships. **^b^** Both Model 1 [Y ~ X + ... (adjusted variables) ] and Model 2 [Y ~ M + X + ... (adjusted variables) ] were utilized for the analysis of indirect effects, with Y indicating depression, X indicating social support or existential well-being, M indicating resilience and adjusted variables including age, living place, physical activity, education level, family income and family relationships. **^c^**The 95% confidence intervals for the indirect effect were calculated using bias-corrected bootstrapping.

## References

[1] Sung H, Ferlay J, Siegel RL, Laversanne M, Soerjomataram I, Jemal A (2021). Global Cancer Statistics 2020: GLOBOCAN Estimates of Incidence and Mortality Worldwide for 36 Cancers in 185 Countries. CA: a cancer journal for clinicians.

[2] Wang YH, Li JQ, Shi JF, Que JY, Liu JJ, Lappin JM (2020). Depression and anxiety in relation to cancer incidence and mortality: a systematic review and meta-analysis of cohort studies. Mol Psychiatry.

[3] Lu D, Andrae B, Valdimarsdottir U, Sundstrom K, Fall K, Sparen P (2019). Psychologic Distress Is Associated with Cancer-Specific Mortality among Patients with Cervical Cancer. Cancer Res.

[4] Herzog TJ, Wright JD (2007). The impact of cervical cancer on quality of life-the components and means for management. Gynecologic oncology.

[5] Richardson GE (2002). The metatheory of resilience and resiliency. Journal of clinical psychology.

[6] Loprinzi CE, Prasad K, Schroeder DR, Sood A (2011). Stress Management and Resilience Training (SMART) program to decrease stress and enhance resilience among breast cancer survivors: a pilot randomized clinical trial. Clin Breast Cancer.

[7] Ye ZJ, Qiu HZ, Liang MZ, Liu ML, Li PF, Chen P (2017). Effect of a mentor-based, supportive-expressive program, Be Resilient to Breast Cancer, on survival in metastatic breast cancer: a randomised, controlled intervention trial. Br J Cancer.

[8] Tamura S, Suzuki K, Ito Y, Fukawa A (2021). Factors related to the resilience and mental health of adult cancer patients: a systematic review. Support Care Cancer.

[9] Shin HW, Noh DY, Lee ES, Nam SJ, Park BW, Ahn SH (2009). Correlates of existential well-being and their association with health-related quality of life in breast cancer survivors compared with the general population. Breast Cancer Res Treat.

[10] Chen W, Chen Y, Xiao H (2022). Existential Distress in Cancer Patients: A Concept Analysis. Cancer nursing.

[11] Peteet JR, Balboni MJ (2013). Spirituality and religion in oncology. CA: a cancer journal for clinicians.

[12] Jimenez-Fonseca P, Lorenzo-Seva U, Ferrando PJ, Carmona-Bayonas A, Beato C, Garcia T (2018). The mediating role of spirituality (meaning, peace, faith) between psychological distress and mental adjustment in cancer patients. Support Care Cancer.

[13] Vos J (2015). Meaning and existential givens in the lives of cancer patients: A philosophical perspective on psycho-oncology. Palliat Support Care.

[14] Usta YY (2012). Importance of social support in cancer patients. Asian Pac J Cancer Prev.

[15] Ikeda A, Kawachi I, Iso H, Iwasaki M, Inoue M, Tsugane S (2013). Social support and cancer incidence and mortality: the JPHC study cohort II. Cancer Causes Control.

[16] Tian X, Jin Y, Chen H, Tang L, Jimenez-Herrera MF (2021). The positive effect of social support on psychological distress among Chinese lung cancer patients: The mediating role of self-esteem. Nurs Open.

[17] Zhao X, Sun M, Yang Y (2021). Effects of social support, hope and resilience on depressive symptoms within 18 months after diagnosis of prostate cancer. Health Qual Life Outcomes.

[18] Ciria-Suarez L, Calderon C, Fernandez Montes A, Antonanzas M, Hernandez R, Rogado J (2021). Optimism and social support as contributing factors to spirituality in Cancer patients. Support Care Cancer.

[19] Yağmur Y, Duman M (2016). The relationship between the social support level perceived by patients with gynecologic cancer and mental adjustment to cancer. Int J Gynaecol Obstet.

[20] Sophie Lebel GMD (2008). Stigma in cancer patients whose behavior may have contributed to their disease. Future Oncology.

[21] Zigmond AS, Snaith RP (1983). The hospital anxiety and depression scale. Acta psychiatrica Scandinavica.

[22] Li Q, Lin Y, Hu C, Xu Y, Zhou H, Yang L (2016). The Chinese version of hospital anxiety and depression scale: Psychometric properties in Chinese cancer patients and their family caregivers. European journal of oncology nursing: the official journal of European Oncology Nursing Society.

[23] Wagnild GM (2009). The Resilience Scale user's guide for the US English version of the Resilience Scale and the 14-Item Resilience Scale (RS-14). Montana: Resilience Center.

[24] Tian J, Hong JS (2013). Validation of the Chinese version of the resilience scale and its cutoff score for detecting low resilience in Chinese cancer patients. Support Care Cancer.

[25] Cohen SR, Mount BM, Strobel MG, Bui F (1995). The McGill Quality of Life Questionnaire: a measure of quality of life appropriate for people with advanced disease. A preliminary study of validity and acceptability. Palliative medicine.

[26] Cui J, Fang F, Shen F, Song L, Zhou L, Ma X (2014). Quality of life in patients with advanced cancer at the end of life as measured by the McGill quality of life questionnaire: a survey in China. J Pain Symptom Manage.

[27] Chen CM (2008). Overview of obesity in Mainland China. Obes Rev.

[28] Preacher KJ, Hayes AF (2004). SPSS and SAS procedures for estimating indirect effects in simple mediation models. Behavior research methods, instruments, & computers: a journal of the Psychonomic Society, Inc.

[29] Hayes AF (2017). Introducation to mediation, moderation, and conditional process analysis: A regression-based approach. New York: Guilford publications.

[30] Zhao Y, Hu B, Liu Q, Wang Y, Zhao Y, Zhu X Social support and sleep quality in patients with stroke: The mediating roles of depression and anxiety symptoms. Int J Nurs Pract. 2021: e12939.

[31] Xu JX, Wu LX, Jiang W, Fan GH (2021). Effect of nursing intervention based on Maslow's hierarchy of needs in patients with coronary heart disease interventional surgery. World J Clin Cases.

[32] Gerino E, Rollè L, Sechi C, Brustia P (2017). Loneliness, Resilience, Mental Health, and Quality of Life in Old Age: A Structural Equation Model. Frontiers in psychology.

[33] Kim SH, Kang S, Kim YM, Kim BG, Seong SJ, Cha SD (2010). Prevalence and predictors of anxiety and depression among cervical cancer survivors in Korea. Int J Gynecol Cancer.

[34] Schaefer SM, Morozink Boylan J, van Reekum CM, Lapate RC, Norris CJ, Ryff CD (2013). Purpose in life predicts better emotional recovery from negative stimuli. PLoS One.

[35] Sharif Nia H, Lehto RH, Seyedfatemi N, Mohammadinezhad M (2021). A path analysis model of spiritual well-being and quality of life in Iranian cancer patients: a mediating role of hope. Support Care Cancer.

[36] Harms CA, Cohen L, Pooley JA, Chambers SK, Galvao DA, Newton RU (2019). Quality of life and psychological distress in cancer survivors: The role of psycho-social resources for resilience. Psychooncology.

[37] Hu T, Xiao J, Peng J, Kuang X, He B (2018). Relationship between resilience, social support as well as anxiety/depression of lung cancer patients: A cross-sectional observation study. J Cancer Res Ther.

[38] Babić R, Babić M, Rastović P, Ćurlin M, Šimić J, Mandić K (2020). Resilience in Health and Illness. Psychiatr Danub.

[39] Sakai A, Terao T, Kawano N, Akase M, Hatano K, Shirahama M (2019). Existential and Mindfulness-Based Intervention to Increase Self-Compassion in Apparently Healthy Subjects (the EXMIND Study): A Randomized Controlled Trial. Front Psychiatry.

